# Post-Translational Modifications in Multiple Myeloma: Mechanisms of Drug Resistance and Therapeutic Opportunities

**DOI:** 10.3390/biom15050702

**Published:** 2025-05-12

**Authors:** Shuoyang Hu, Jirun Xu, Weiyan Cui, Haoran Jin, Xiaoyu Wang, Yasen Maimaitiyiming

**Affiliations:** 1 Department of Immunology, School of Basic Medical Sciences, Xinjiang Medical University, Urumqi 830011, China; husy1375@stu.xjmu.edu.cn (S.H.); 17799768791@stu.xjmu.edu.cn (J.X.); cuiweiyan@stu.xjmu.edu.cn (W.C.); 18755666782@stu.xjmu.edu.cn (H.J.); wxy106@stu.xjmu.edu.cn (X.W.); 2 Xinjiang Key Laboratory of Molecular Biology for Endemic Diseases, Xinjiang Medical University, Urumqi 830011, China

**Keywords:** multiple myeloma, post-translational modifications, drug resistance, signaling pathways, tumor microenvironment, proteasome inhibitors (PIs), immunomodulatory drugs (IMiDs), therapeutic targets

## Abstract

Multiple myeloma (MM) remains an incurable hematologic malignancy due to the inevitable development of drug resistance, particularly in relapsed or refractory cases. Post-translational modifications (PTMs), including phosphorylation, ubiquitination, acetylation, and glycosylation, play pivotal roles in regulating protein function, stability, and interactions, thereby influencing MM pathogenesis and therapeutic resistance. This review comprehensively explores the mechanisms by which dysregulated PTMs contribute to drug resistance in MM, focusing on their impact on key signaling pathways, metabolic reprogramming, and the tumor microenvironment. We highlight how PTMs modulate drug uptake, alter drug targets, and regulate cell survival signals, ultimately promoting resistance to PIs, IMiDs, and other therapeutic agents. Furthermore, we discuss emerging therapeutic strategies targeting PTM-related pathways, which offer promising avenues for overcoming resistance to treatment. By integrating preclinical and clinical insights, this review underscores the potential of PTM-targeted therapies to enhance treatment efficacy and improve patient outcomes in MM.

## 1. Introduction

Multiple myeloma (MM) is a hematologic malignancy characterized by the clonal expansion of plasma cells within the bone marrow, leading to the excessive production of monoclonal immunoglobulins [1]. This abnormal proliferation of plasma cells results in a cascade of clinical manifestations, including hypercalcemia, renal impairment, anemia, and osteolytic bone lesions, commonly referred to as the CRAB criteria (calcium elevation, renal failure, anemia, and bone lesions) [2]. Despite significant advancements in MM therapy, the disease remains incurable due to the emergence of therapeutic resistance, particularly in patients with relapsed or refractory MM (RRMM) [3]. Consequently, understanding the molecular mechanisms of RRMM and developing novel therapeutic strategies to overcome resistance is critical to improving patient outcomes.

Traditional treatment options for MM include chemotherapy, proteasome inhibitors (PIs), immunomodulatory drugs (IMiDs), and autologous hematopoietic stem cell transplantation (ASCT). Alkylating agents like melphalan and cyclophosphamide disrupt DNA replication, leading to tumor cell death [4,5]. PIs such as bortezomib and carfilzomib prevent protein degradation in MM cells, inducing apoptosis [6,7,8]. IMiDs, like lenalidomide and pomalidomide, boost immune-mediated destruction of myeloma cells while modulating tumor microenvironments [9]; ASCT is employed for eligible patients (particularly younger individuals with adequate organ function) by harvesting the patient’s own hematopoietic stem cells, administering high-dose melphalan to eliminate residual myeloma cells, and then reinfusing the stem cells to restore bone marrow function [10]. These approaches have extended patient survival, but most patients eventually develop resistance or relapse after prolonged use.

Recent advances in MM treatment have introduced innovative therapies such as monoclonal antibodies (mAbs), chimeric antigen receptor T-cell (CAR-T) therapy, and bispecific T-cell engagers (BiTEs). CAR-T therapy, targeting B-cell maturation antigen (BCMA), has demonstrated remarkable efficacy in RRMM patients [11,12]. Monoclonal antibodies like daratumumab, targeting CD38, have significantly improved outcomes when combined with traditional therapies [13]. Meanwhile, BiTEs are showing promise by bridging T-cells to myeloma cells, enhancing immune-mediated destruction [14]. Although these treatments represent groundbreaking advancements, challenges like accessibility and resistance remain, highlighting the need for further research to refine these therapies and enhance patient outcomes.

Post-translational modifications (PTMs) are crucial regulators of cellular processes, playing key roles in protein function, stability, localization, and interactions. In the context of MM, the dysregulation of PTMs has been implicated in both disease pathogenesis and therapeutic resistance [15,16]. PTMs, such as phosphorylation, acetylation, and ubiquitination, are involved in the regulation of tumor suppressor proteins and oncogenes, contributing to tumor progression, metastasis, and drug resistance. Thus, understanding the interplay between PTMs and therapeutic resistance offers a promising avenue for the development of novel combination therapies to improve patient outcomes.

Roles of histone modifications on MM drug resistance have been extensively reviewed elsewhere [17,18]. Here, we mainly focus on recent advances in the study of PTMs and their contribution to therapeutic resistance in MM, with an emphasis on identifying novel therapeutic targets and strategies to improve patient outcomes.

## 2. Major PTMs Implicated in MM Pathogenesis and Progression

Proteins, biomacromolecules that are synthesized by ribosomes and subsequently processed by the endoplasmic reticulum (ER) and Golgi apparatus, play essential roles in cellular functions. Throughout their lifecycle, proteins undergo various PTMs, which typically involve the covalent attachment of small molecular groups to specific amino acid residues under enzymatic catalysis [19,20]. Common modifications implicated in MM include phosphorylation, ubiquitination, methylation, acetylation, SUMOylation (modification by small ubiquitin-related modifier), and glycosylation, often occurring at amino acid residues such as lysine, serine, threonine, arginine, and tyrosine [2]. Studies have shown that PTMs are functionally diverse and closely related to the onset and progression of numerous diseases, including MM, neurological disorders, immune diseases, and cardiovascular diseases [21,22,23].

**Figure 1:**
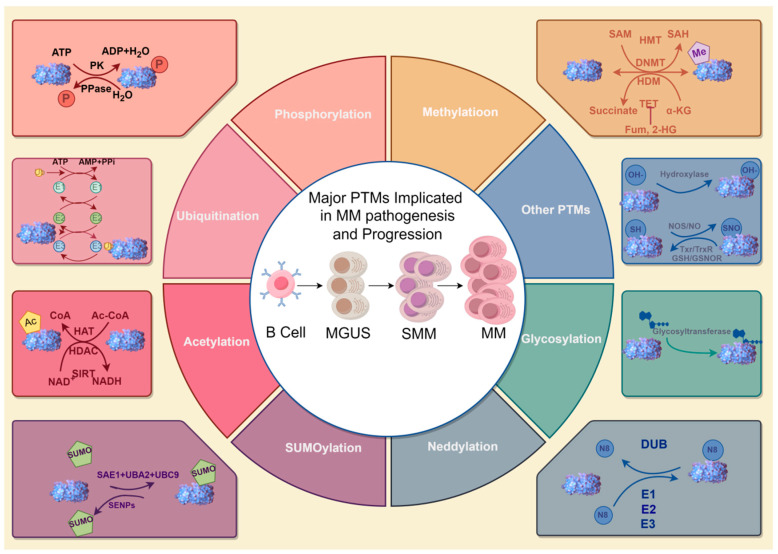
A summary of major PTMs implicated in MM pathogenesis and progression. The center illustrates the clinical progression stages of MM (MGUS → SMM → MM), defined by diagnostic criteria including monoclonal protein levels, bone marrow plasma cell infiltration, and CRAB symptoms. The surrounding circular diagrams depict key PTMs (e.g., phosphorylation, SUMOylation) that drive molecular pathogenesis across disease stages. While no specific PTM is exclusively linked to a single clinical stage, dysregulated PTMs collectively contribute to malignant transformation and drug resistance during progression. Abbreviations: MGUS, Monoclonal Gammopathy of Undetermined Significance; SMM, Smoldering MM; MM, MM; ATP, Adenosine Triphosphate; ADP, Adenosine Diphosphate; PK, Pyruvate Kinase; PPase, Pyrophosphatase; SAM, S-Adenosylmethionine; HMT, Histone Methyltransferase; SAH, S-Adenosylhomocysteine; DNMT, DNA Methyltransferase; MD, Methyltransferase Domain; HDM, Histone Demethylase; TET, Ten-Eleven Translocation methylcytosine dioxygenase; α-KG, Alpha-Ketoglutarate; Fum, Fumarate; 2-HG, 2-Hydroxyglutarate; AMP, Adenosine Monophosphate; PPI, Inorganic Pyrophosphate; NOS, Nitric Oxide Synthase; NO, Nitric Oxide; SNO, S-Nitrosothiol; GSH, Glutathione; GSNOR, Glutathione-Dependent Formaldehyde Dehydrogenase; CoA, Coenzyme A; Ac-CoA, Acetyl-Coenzyme A; HAT, Histone Acetyltransferase; ADA, Adenosine Deaminase; NADH, Nicotinamide Adenine Dinucleotide (Reduced Form); SAE1, SUMO Activating Enzyme Subunit 1; UBA2, Ubiquitin-like Modifier Activating Enzyme 2; UBC9, Ubiquitin Conjugating Enzyme 9; SENPs, Sentrin/SUMO-specific Proteases; DUB, Deubiquitinating Enzyme; E1, Ubiquitin-Activating Enzyme; E2, Ubiquitin-Conjugating Enzyme; E3, Ubiquitin Ligase. The figure was created with Figdraw. Hu, S. (2025) https://www.figdraw.com/ (accessed on 19 March 2025).

Generally, PTMs are installed on amino acid residues by enzymes that could be termed “writers”, and can be removed by another group of enzymes termed “erasers” [24]. Such dynamic modifications exert a multitude of functions, as mentioned above. In this part, we recapitulate PTMs implicated in the pathogenesis and progression of MM (Figure 1).

### 2.1. Phosphorylation

Phosphorylation involves the covalent attachment of a phosphate group to an amino acid residue under the catalysis of protein kinases [25,26]. Protein phosphatases are responsible for removing the phosphate group from proteins. It is one of the most abundant and common PTMs in eukaryotes and plays a crucial role in intracellular signal transduction, regulating various biological processes such as the cell cycle, differentiation, transformation, development, and peptide hormone responses [27]. Phosphorylation plays a crucial role in the pathogenesis and progression of MM. It is involved in the regulation of various signaling pathways that drive the proliferation, survival, and metastasis of MM cells. For instance, the phosphorylation of signal transducer and activator of transcription 3 (STAT3) is a key event in MM progression. STAT3 activation, often mediated by phosphorylation at Tyr705, promotes cell proliferation, survival, and immune evasion in MM cells [28,29].

Moreover, phosphorylation events are implicated in the regulation of metabolic reprogramming in MM. For example, the phosphorylation of AMP-activated protein kinase (AMPK) and mammalian target of rapamycin (mTOR) influences the metabolic state of MM cells, contributing to their survival and progression [30,31]. Phosphorylation of histone proteins and other epigenetic regulators also plays a role in the transcriptional dysregulation observed in MM [32,33]. Phosphorylation-mediated signaling pathways and metabolic reprogramming are central to the pathogenesis and progression of MM, highlighting the importance of targeting these pathways for therapeutic intervention.

### 2.2. Acetylation

Acetylation is a process involving the attachment of an acetyl group (-COCH3) to a protein molecule, which includes two types: N-terminal acetylation (Nt-acetylation) and internal acetylation. N-terminal acetylation refers to the covalent modification on the α-amino group of the first amino acid residue at the N-terminus of the protein, while internal acetylation occurs on the ε-amino group of lysine residues within proteins [34]. This acetylation modification is mainly catalyzed by two types of enzymes: histone acetyltransferases (HATs) are responsible for adding acetyl groups, while histone deacetylases (HDACs) are responsible for their removal [35]. Non-histone protein acetylation plays a significant role in the pathogenesis and progression of MM. For instance, the acetylation of APE1 (apurinic/apyrimidinic endonuclease 1) at lysine residues K6 and K7 has been shown to regulate MDR1 (multidrug resistance protein 1) expression, thereby influencing drug efflux and resistance to alkylating agents like melphalan [36]. Additionally, the ADA2B subunit of the SAGA complex regulates MYC(c-Myc) expression through acetylation, which is crucial for maintaining oncogenic programs in MM [37]. KDM6A regulates immune recognition genes in MM through H3K27 acetylation, highlighting its role in immune evasion [38,39]. Furthermore, metabolic regulation through non-histone protein acetylation is also a key factor. It was found that ENO1, a MYC target gene, promotes MM progression by enhancing mitophagy through YWHAZ acetylation, contributing to metabolic regulation [40,41,42]. These findings highlight the importance of non-histone protein acetylation in driving MM progression.

### 2.3. Ubiquitination

Ubiquitination is a widespread protein modification in eukaryotes that involves the attachment of ubiquitin, a highly conserved protein composed of 76 amino acids, to target proteins. This modification is catalyzed by the E1 ubiquitin-activating enzyme, E2 ubiquitin-conjugating enzymes, and E3 ubiquitin ligases. Erasers of ubiquitination include a broad range of deubiquitinating enzymes, namely ubiquitin-specific proteases (USPs) and ubiquitin C-terminal hydrolases (UCHs) [43,44]. Ubiquitination serves as a dynamic regulatory switch, controlling protein stability, subcellular localization, activity, and protein-protein interactions [45,46,47]. In the context of MM, dysregulated ubiquitination not only drives oncogenic signaling and disease progression but also profoundly impacts therapeutic resistance.

In MM, the ubiquitin-proteasome system (UPS) is critical for maintaining protein homeostasis. MM cells rely heavily on the UPS to degrade excess monoclonal immunoglobulins and misfolded proteins generated by their high proliferative rate [16]. However, dysfunction of the UPS can lead to proteotoxic stress, which paradoxically contributes to disease progression by activating compensatory survival pathways (e.g., NF-κB and HSF1) [16,48]. For example, the E3 ubiquitin ligase CRL4CRBN is hijacked by IMiDs (lenalidomide/pomalidomide) to degrade IKZF1/3, key transcription factors sustaining MM cell survival [48]. Conversely, mutations or downregulation of CRBN disrupt IMiD-induced degradation, representing a primary mechanism of IMiD resistance [49].

Beyond its role in protein degradation, ubiquitination dynamically modulates signaling pathways that drive MM pathogenesis. For instance, TRAF6-mediated K63-linked ubiquitination of AKT enhances its activation, promoting PI3K/AKT/mTOR signaling and cell survival [50]. Additionally, deubiquitinating enzymes (DUBs) such as USP7 and USP14 counteract ubiquitination, stabilizing oncoproteins (e.g., Mcl-1, NEK2) and contributing to resistance against proteasome inhibitors like bortezomib [51,52]. These DUBs also protect MM cells from ER stress-induced apoptosis by deubiquitinating key sensors like IRE1α, disrupting the UPR pathway [53].

Overall, ubiquitination acts as a double-edged sword in MM: while it maintains protein quality control and regulates physiological signaling, its dysregulation underpins both disease progression and therapeutic evasion. Targeting aberrant ubiquitination pathways (e.g., E3 ligases, DUBs) has emerged as a promising strategy to overcome resistance and restore treatment sensitivity.

### 2.4. Methylation

Methylation is the process of introducing methyl groups into proteins, which can occur on various amino acids such as arginine, lysine, and histidine. Methylation is involved in regulating protein-protein interactions, thereby exerting a significant impact on a series of crucial cellular events, such as transcriptional stress response, aging, protein repair, and neuronal differentiation [54]. Nonhistone protein methylation plays a crucial role in the development and progression of MM by modulating the function of transcription factors, signaling molecules, and other regulatory proteins. For example, the histone methyltransferase MMSET (also known as NSD2) is frequently overexpressed in MM due to the t(4;14) chromosomal translocation. MMSET catalyzes the methylation of histone H3 lysine 36 (H3K36me2), leading to global changes in histone methylation patterns and chromatin structure [55,56]. This aberrant methylation can dysregulate genes involved in cell cycle control, apoptosis, and DNA repair [57]. Promoter methylation of the ABCG2 gene, which encodes an ATP-binding cassette transporter, enhances drug efflux capacity by upregulating its expression, contributing to chemoresistance against agents such as topotecan and doxorubicin [58,59].

Another important example is the methylation of the suppressor of cytokine signaling 1 (SOCS1), a key regulator of cytokine signaling and immune response. In MM, SOCS1 methylation is often associated with increased resistance to apoptosis and enhanced cell survival. Studies have shown that demethylation agents, such as 5-azacytidine, can reverse SOCS1 methylation, leading to its reactivation and increased sensitivity of MM cells to apoptosis [60]. Additionally, methylation of nonhistone proteins can affect the interaction between myeloma cells and the bone marrow microenvironment, which is crucial for disease progression [61].

### 2.5. SUMOylation

SUMOylation is the process of covalently attaching small ubiquitin-like modifier (SUMO) proteins to target proteins as a post-translational modification. This process is mediated by specific enzymes known as SUMOylation writers and erasers [62,63]. Writers include SUMO E1-activating enzyme (SAE1/SAE2), SUMO E2-conjugating enzyme (UBC9), and various SUMO E3 ligases (such as PIAS proteins), which facilitate the attachment of SUMO to target proteins. On the other hand, erasers are SUMO-specific proteases (SENPs), which remove SUMO from modified proteins, thus reversing the modification [64]. Abnormalities in SUMOylation can affect the sensitivity and drug resistance of MM cells through various mechanisms. For instance, SUMOylation of β-catenin is associated with poor prognosis in MM. Inhibiting SUMOylation downregulates β-catenin levels by promoting its ubiquitin-proteasomal degradation, thereby suppressing the Wnt/β-catenin signaling pathway [65]. This suggests that SUMOylation of β-catenin contributes to the aberrant proliferation of MM cells.

Additionally, the SUMOylation pathway is significantly enhanced in MM patients compared to normal plasma cells. High expression of SUMOylation-related enzymes, such as UBE2I and PIAS1, is associated with poor survival outcomes. This indicates that dysregulated SUMOylation is a critical factor in MM progression and may serve as a potential therapeutic target [62]. Moreover, SUMOylation can regulate protein stability in MM cells. For example, SUMOylation of Rep78, a regulatory protein, affects its stability and function, suggesting that SUMOylation may impact viral replication and MM cell survival [66].

### 2.6. Neddylation

Neddylation is a post-translational modification that involves the covalent attachment of the ubiquitin-like protein NEDD8 to substrate proteins, particularly cullins, which are components of Cullin-RING Ligases (CRLs) [67,68,69]. CRLs are the largest family of E3 ubiquitin ligases and play a critical role in regulating protein degradation through the UPS. Aberrant regulation of this pathway has been implicated in the pathogenesis of MM [68,69]. Dysregulation of the neddylation pathway can lead to imbalances in protein homeostasis, which is essential for maintaining cellular functions. This disruption is particularly relevant in MM, where the UPS is already compromised due to the high protein synthesis load of malignant plasma cells [70]. Neddylation is also involved in the activation of various oncogenic pathways in MM. For example, the neddylation of cullins is essential for the function of CRLs, which regulate the degradation of key proteins involved in cell cycle progression, apoptosis, and DNA repair. Overactivation of these pathways can contribute to tumor growth and progression [69,70]. The bone marrow microenvironment in MM is highly immunosuppressive, and neddylation may play a role in modulating this environment. Studies have shown that inhibition of neddylation can enhance the expression of NK cell-activating ligands (e.g., MICA and MICB) on MM cells, making them more susceptible to immune-mediated killing [71].

### 2.7. Glycosylation

Glycosylation, the enzymatic process of attaching sugar moieties (glycans) to proteins or lipids, is a critical post-translational modification that influences protein folding, stability, and function. This process occurs in two primary forms: N-glycosylation, where glycans are attached to the nitrogen atom of asparagine side chains [72,73], and O-glycosylation, where glycans are linked to the oxygen atom of serine or threonine residues [74,75]. Both N-glycosylation and O-glycosylation play significant roles in the pathogenesis of MM, affecting cell signaling, immune evasion, and disease progression. Studies have shown that monoclonal immunoglobulins produced by MM cells often exhibit altered glycosylation patterns compared to normal immunoglobulins. For instance, a decrease in galactosylation and sialylation, along with an increase in fucosylation, is commonly observed in MM patients [73,76]. These changes are present across all International Staging System (ISS) stages, including low-risk ISS I, indicating that glycosylation alterations are an early event in MM development. Abnormal glycosylation of immunoglobulins has also been linked to bone loss in MM. Specifically, reduced galactosylation and sialylation of IgG in MM patients with bone disease suggest that glycosylation status can influence the interaction between MM cells and the bone microenvironment [77]. This finding highlights the potential role of glycosylation in promoting osteoclast differentiation and bone resorption.

Glycosylation changes in MM are not static and can evolve with disease progression. For example, difucosylation of diantennary glycans decreases as MM progresses, indicating that glycosylation profiles may serve as biomarkers for disease stage and progression [73]. Additionally, glycosylation alterations in the immunoglobulin variable region (Fab) have been identified as potential risk factors for disease progression in plasma cell disorders [72]. Genome-wide association studies (GWAS) have identified loci associated with IgG glycosylation that also show pleiotropy with autoimmune diseases and hematological cancers, including MM [78]. These genetic insights suggest that glycosylation pathways are intricately linked to the molecular mechanisms underlying MM pathogenesis.

### 2.8. Other PTMs

Hydroxylation and nitrosylation are emerging as important PTMs involved in the pathogenesis and progression of MM. Hydroxylation, catalyzed by enzymes such as prolyl hydroxylases, plays a role in stabilizing hypoxia-inducible factor-1 alpha (HIF-1α), which promotes tumor adaptation to hypoxic conditions [79,80]. In MM, the methylation of EGLN3, a prolyl hydroxylase, is associated with poor prognosis and advanced disease stages, suggesting that hydroxylation may contribute to disease progression [79]. Nitrosylation, specifically S-nitrosylation, has also been implicated in MM. This modification can inhibit the activity of key signaling pathways such as STAT3 and NF-κB, which are often hyperactivated in MM cells [81]. It was shown that S-nitrosothiols, such as S-nitroso-N-acetylcysteine (SNAC), can inhibit MM cell proliferation and survival by targeting these pathways [81]. This suggests that nitrosylation may be a potential therapeutic target for overcoming drug resistance and improving patient outcomes.

Collectively, various PTMs are critical for regulating cellular processes and maintaining cell function, playing a particularly significant role in the pathogenesis, progression and drug resistance of MM (Table 1). Understanding these modifications is essential for identifying new therapeutic targets and developing more effective treatments for MM.

**Table 1:** Comprehensive Summary of PTM Types, Key Proteins, and Mechanisms in MM Pathogenesis and Drug Resistance.

PTM Type	Key Proteins/Molecules	Mechanism in MM	References
**Phosphorylation**			
	STAT3 (Tyr705)	Phosphorylated STAT3 activates NF-κB/STAT3 signaling, promoting proliferation, survival, and immune evasion.	[28,29]
	AMPK/mTOR	Phosphorylation regulates metabolic reprogramming; hyperactivation supports MM survival under stress.	[30,82]
	MAFb	GSK3-mediated phosphorylation stabilizes MAFb, disrupting proteasome inhibitor targets and inducing resistance.	[83]
	Akt/GSK-3β	Akt phosphorylates and inactivates GSK-3β, stabilizing c-Myc to drive drug resistance.	[84]
	MKK4/7-JNK	GCK-induced phosphorylation activates RAS-mutant MAPK signaling, promoting adaptive resistance.	[29]
**Acetylation**			
	ADA2B (SAGA complex)	Acetylates histones to regulate c-Myc expression, sustaining oncogenic programs.	[37]
	KDM6A	Modulates H3K27 acetylation to suppress immune recognition genes, enabling immune evasion.	[38,39]
	YWHAZ	Acetylation by ENO1 enhances mitophagy, supporting metabolic adaptation in MM progression.	[40]
	APE1 (K6/K7)	Acetylation enhances DNA repair via BER and upregulates MDR1, promoting melphalan resistance.	[36]
**Ubiquitination**			
	CRL4CRBN	IMiDs recruit CRBN to ubiquitinate IKZF1/3 for proteasomal degradation; mutations confer resistance.	[48]
	IKZF1/IKZF3	Degradation by IMiDs suppresses MM survival; loss of ubiquitination leads to IMiD resistance.	[85,86]
	HMGB1	MALAT-1-induced ubiquitination promotes autophagy and inhibits apoptosis, driving drug resistance.	[87]
	BRCC36	Cleaves K63-ubiquitin chains on CRBN, stabilizing it to enhance IMiD sensitivity.	[49]
	NEK2/USP7/TRIP13	USP7 deubiquitinates NEK2, stabilizing it to promote chromosomal instability and PI resistance.	[51,88]
**Methylation**			
	MMSET (NSD2)	Catalyzes H3K36me2, dysregulating tumor suppressor genes and promoting MM progression.	[55,56]
	SOCS1	Methylation silences SOCS1, enhancing cytokine signaling and resistance to apoptosis.	[60]
	EGLN3	Methylation of EGLN3 (prolyl hydroxylase) correlates with hypoxia adaptation and poor prognosis.	[79]
**SUMOylation**			
	β-catenin	SUMOylation stabilizes β-catenin, activating Wnt signaling to drive proliferation and drug resistance.	[65]
	IκBα	SENP2 deficiency increases SUMOylation of IκBα, activating NF-κB and bortezomib resistance.	[89]
	IRF4/c-Myc	SUMOylation stabilizes IRF4/c-Myc; inhibition by TAK-981 restores lenalidomide sensitivity.	[90]
**Neddylation**			
	CRLs (Cullin-RING ligases)	Neddylation activates CRLs; inhibition by MLN4924 stabilizes pro-apoptotic proteins (e.g., NOXA).	[69,91]
	REDD1	Neddylation blockade stabilizes REDD1, inhibiting PI3K/AKT/mTOR and overcoming resistance.	[91]
**Glycosylation**			
	α4β1/α4β7 integrins	Sialylation enhances adhesion to bone marrow stroma, promoting CAM-DR and drug sanctuary.	[92,93,94]
	CD38/PSGL-1	Sialylation masks CD38 epitopes (daratumumab resistance); PSGL-1 binds Siglec-7 to suppress NK activity.	[76,93]
	IgG (Fab region)	Altered glycosylation in the Fab region correlates with disease progression and bone loss.	[72,78]
**Hydroxylation**			
	HIF-1α	Hydroxylation stabilizes HIF-1α under hypoxia, promoting tumor adaptation and survival.	[79]
**Nitrosylation**			
	STAT3/NF-κB	S-nitrosylation inhibits STAT3/NF-κB activity; SNAC reverses hyperactivation to restore drug sensitivity.	[81]
**Deubiquitination**			
	USP14/UCHL5	Deubiquitinate misfolded proteins to reduce proteotoxic stress, conferring PI resistance.	[53]
**Deacetylation**			
	HDAC1	Deacetylates histones and non-histones (e.g., HPV), enhancing DNA repair and resistance to DNA damage.	[95]

## 3. PTM Targeting Therapies in MM

Targeting PTMs of proteins has emerged as a promising therapeutic strategy in the treatment of MM. Several classes of drugs that modulate PTMs are currently in clinical use or under investigation, offering new avenues for more effective MM management.

### 3.1. PIs

PIs are a cornerstone of MM therapy, targeting the UPS, a critical PTM pathway responsible for protein degradation. By inhibiting the proteasome, these drugs induce the accumulation of ubiquitinated proteins, leading to ER stress, activation of the unfolded protein response (UPR), and ultimately apoptosis in MM cells [45,96,97]. The first-in-class proteasome inhibitor, bortezomib, has significantly improved outcomes in both newly diagnosed and relapsed/refractory MM. It targets the 26S proteasome, leading to the accumulation of pro-apoptotic proteins such as NOXA and BIM, and is often used in combination with dexamethasone and other agents. Its subcutaneous administration has reduced the risk of peripheral neuropathy. Carfilzomib, the second-generation proteasome inhibitor, offers improved specificity for the proteasome’s chymotrypsin-like activity and reduced neurotoxicity compared to bortezomib [98,99,100,101,102]. It is approved for RRMM and is often used in combination with dexamethasone and lenalidomide. Ixazomib, the first oral proteasome inhibitor, provides convenience and improved patient adherence [103]. It is approved for RRMM in combination with lenalidomide and dexamethasone, offering a well-tolerated option for maintenance therapy. Together, these agents have revolutionized MM treatment, significantly improving overall survival (OS) and progression-free survival (PFS) by disrupting protein homeostasis, a critical vulnerability in MM cells.

### 3.2. IMiDs

While not direct PTM-targeting drugs, IMiDs modulate PTMs by recruiting specific substrates to the cereblon E3 ubiquitin ligase complex, leading to their ubiquitination and degradation [104]. This mechanism disrupts MM cell survival pathways and enhances immune-mediated tumor cell killing. Lenalidomide, approved for both newly diagnosed and relapsed/refractory MM, induces the degradation of IKZF1 and IKZF3, transcription factors critical for MM cell survival [85,86]. It is often used in combination with dexamethasone and PIs and is a mainstay of maintenance therapy. Pomalidomide, a more potent IMiD, is approved for RRMM and has shown efficacy in patients refractory to lenalidomide and bortezomib, making it a valuable option for heavily pretreated patients [105,106]. IMiDs have transformed MM treatment, with lenalidomide-based regimens significantly improving OS and PFS. Their ability to modulate PTMs through the cereblon pathway has made them a cornerstone of MM therapy, offering durable responses and improved outcomes across all stages of the disease.

### 3.3. Histone Deacetylase (HDAC) Inhibitors

HDAC inhibitors target epigenetic modifications by inhibiting the deacetylation of histones and non-histone proteins, leading to the reactivation of tumor suppressor genes, disruption of MM cell proliferation, and induction of apoptosis [107]. Panobinostat is the first and currently the only FDA-approved HDAC inhibitor for MM, though its approval status may have evolved in recent years [108]. Approved for RRMM in combination with bortezomib and dexamethasone, panobinostat has shown efficacy in patients who have become refractory to PIs and IMiDs, highlighting its role in overcoming drug resistance. It enhances the activity of PIs by further disrupting protein homeostasis, making it a valuable addition to combination therapies [109,110].

## 4. Role of PTMs in Drug Resistance Mechanisms in MM

**Figure 2:**
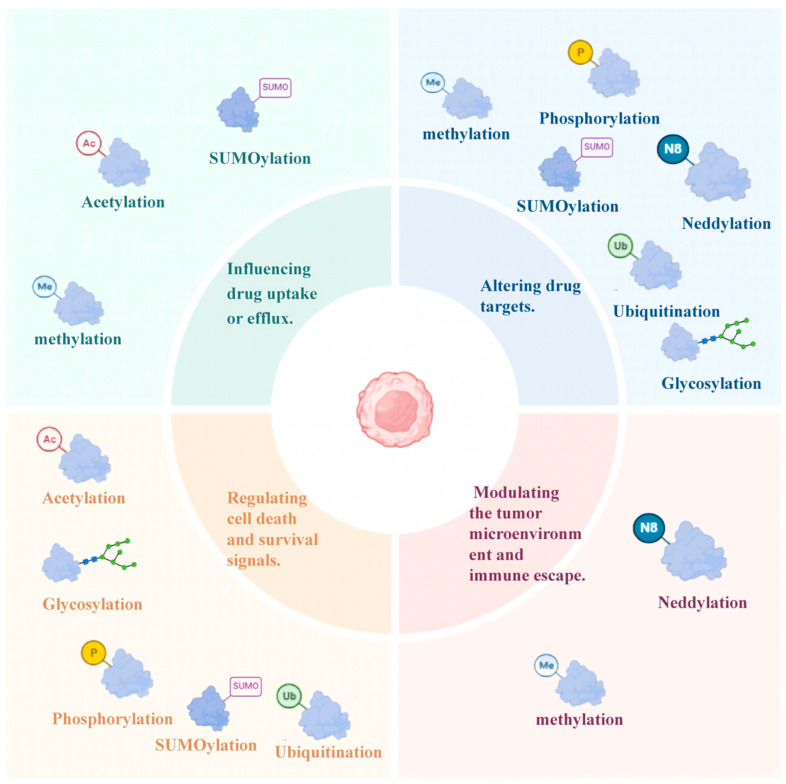
Four Major Mechanisms of PTM-Mediated Drug Resistance in MM. This figure summarizes the key PTMs involved in the drug resistance of MM, organized into four functional categories (distinguished by colored background blocks) and their corresponding PTMs (annotated with colored icons and abbreviations). The figure was created in Biorender. Hu, S. (2025) https://BioRender.com (accessed on 19 March 2025).

PTMs play a pivotal role in the pathogenesis and progression of MM, particularly in the development of drug resistance. These modifications, including phosphorylation, methylation, acetylation, and ubiquitination, among others, regulate key cellular processes such as protein function, stability, and interactions. In the context of drug resistance, PTMs can alter drug uptake, efflux, target activity, and cell survival pathways, ultimately enabling MM cells to evade therapeutic interventions (Figure 2). Understanding the mechanisms by which PTMs contribute to drug resistance is crucial for developing targeted strategies to overcome treatment challenges in MM.

### 4.1. Influencing Drug Uptake or Efflux

#### 4.1.1. SUMOylation

**Figure 3:**
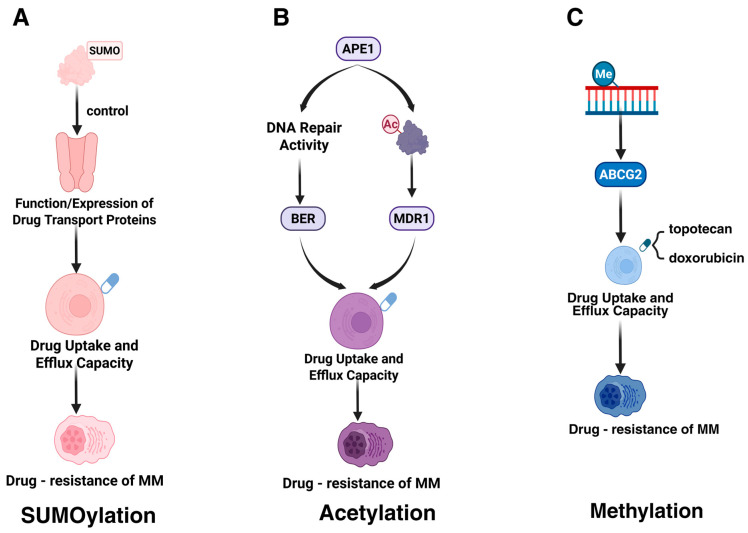
PTMs affecting drug uptake or efflux. This figure summarizes the impact of three PTMs on drug uptake or efflux capacity and their association with drug resistance in MM. (**A**) SUMOylation regulates the function or expression of drug transport proteins, affecting drug uptake and efflux, leading to MM drug resistance. (**B**) Acetylation modification of the APE1 protein may enhance DNA repair activity through the base excision repair (BER) pathway, thereby altering drug accumulation within cells and promoting MM drug resistance. (**C**) Methylation modification of the ABCG2 protein is associated with the efflux capacity of drugs such as topotecan and doxorubicin, further exacerbating MM drug resistance. The figure was created in Biorender. Hu, S. (2025) https://BioRender.com (accessed on 19 March 2025).

A study conducted by Heynen et al. highlights the correlation between an overactive SUMO signaling pathway and resistance to carfilzomib, a proteasome inhibitor utilized in MM therapy. SUMOylation, a type of PTM, can modulate the function or expression of drug transporter proteins, thereby potentially altering their capacity to manage drug uptake and efflux (Figure 3A). This modification might decrease the intracellular concentration of therapeutic agents, consequently diminishing their efficacy [16,111]. Gaining insights into how SUMOylation influences the activity of drug transporter proteins is crucial for devising strategies aimed at enhancing drug accumulation within drug-resistant myeloma cells.

#### 4.1.2. Acetylation

The acetylation modification of APE1 (specifically at lysine residues K6 and K7) enhances cellular resistance to melphalan by regulating the expression of multidrug resistance protein 1 (MDR1), thereby promoting drug efflux (Figure 3B) [36]. Additionally, the DNA repair activity of APE1 mediates resistance through the base excision repair (BER) pathway, which repairs melphalan-induced DNA damage [36].

#### 4.1.3. Methylation

The methylation modification of the ABCG2 promoter enhances drug efflux capacity by regulating the expression level of the ABCG2 gene, thereby influencing drug resistance in MM [58,59]. High expression of ABCG2, functioning as an ATP-binding cassette (ABC) transporter, mediates the active efflux of chemotherapeutic agents (e.g., topotecan, doxorubicin), reducing intracellular drug concentrations (Figure 3C). Additionally, exposure to chemotherapeutic drugs can further upregulate ABCG2 expression, aggravating drug resistance [112].

### 4.2. Altering Drug Targets

#### 4.2.1. Phosphorylation

**Figure 4:**
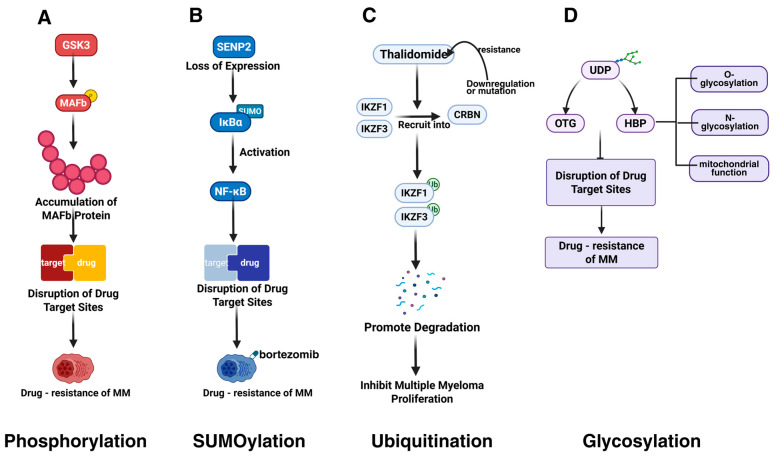
PTMs altering drug targets in MM. This figure illustrates four key PTMs that disrupt drug target sites and contribute to drug resistance in MM. (**A**) GSK3-mediated phosphorylation stabilizes MAPb protein, destabilizing drug targets and promoting resistance. (**B**) Loss of SENP2 triggers IκBα SUMOylation, activating NF-κB signaling and enhancing resistance. (**C**) CRBN downregulation or mutations cause IMiD resistance. (**D**) Upregulated mitochondrial biogenesis markers (PGC1α, SIRT1), enhanced antioxidant capacity, and maintained mitochondrial integrity reduce apoptosis and promote resistance. The figure was created in Biorender. Hu, S. (2025) https://BioRender.com (accessed on 19 March 2025).

Qiang et al. discovered that the MAFb protein is overexpressed in MM cells as a result of the t(14;20) translocation or Igλ insertion, with its protein levels being regulated by PTMs (Figure 4A). GSK3 is capable of phosphorylating the MAFb protein, thereby controlling its degradation. PIs can impede the degradation of the MAFb protein, leading to its accumulation in both the nucleus and cytoplasm [83]. This accumulation can disrupt drug targets and contribute to cellular resistance against PIs. GCK promotes the proliferation and survival of RAS-mutant MM cells by inducing phosphorylation-mediated activation of the MKK4/7-JNK signaling pathway. Inhibiting GCK blocks this phosphorylation process, leading to the degradation of key transcription factors (e.g., IKZF1/3, c-MYC), thereby overcoming resistance to PIs (e.g., bortezomib) and IMiDs [29]. Akt-mediated phosphorylation of GSK-3β (p-GSK-3β) suppresses its kinase activity, impairing its ability to degrade c-Myc. This results in c-Myc protein stabilization and accumulation, promoting MM cell survival and drug resistance [84].

#### 4.2.2. SUMOylation

In MM, the overexpression of the SUMO E2 conjugating enzyme Ubc9, the SUMO E3 ligase PIAS1, and the SUMOylation-inducing factor, the tumor suppressor ARF, is associated with poor prognosis [113]. Ubc9, a central protein in SUMOylation, may modulate drug resistance by altering the properties of drug targets [63]. Another study found that deficiency in SENP2 expression leads to increased SUMOylation of IκBα and subsequent NF-κB activation, which can induce bortezomib resistance (Figure 4B) [89]. This resistance may be mediated by changes in the activity or expression levels of drug targets.

Van Andel et al. found that the deletion of the tumor suppressor CYLD, overexpression of the transcriptional co-activator BCL9, and SUMOylation of β-catenin can abnormally activate the Wnt signaling pathway, potentially altering drug target activity or expression levels [114]. Alterations of SUMOylation also affect the resistance of MM to lenalidomide. The lenalidomide-resistant MMR10R cell line shows higher levels of SUMO E1 (SAE2) and overall SUMOylation compared to the lenalidomide-sensitive MM1S cell line [90]. Inhibition of SUMOylation with the SUMO E1 inhibitor TAK-981 significantly increases myeloma cell sensitivity to lenalidomide. SUMOylation may also modulate drug sensitivity by regulating the IRF4-Myc pathway, with lenalidomide resistance associated with overexpression of IRF4 and c-Myc [90]. TAK-981 can reduce IRF4 and c-Myc expression, affecting drug targets and resistance by decreasing DOT1L and histone H3 lysine 79 methylation, reducing IRF4 gene transcription, and enhancing IRF4 protein degradation.

#### 4.2.3. Ubiquitination

BRCC36, an interacting protein of CRBN (a substrate receptor of cullin 4-RING E3 ligase (CRL4)), is considered a primary target of the immunomodulatory drug thalidomide. Thalidomide and its structural analogs lenalidomide and pomalidomide promote the ubiquitination-mediated degradation of two essential transcription factors, IKZF1 (Ikaros) and IKZF3 (Aiolos), by directly recruiting them to CRBN, thereby inhibiting the proliferation of myeloma cells. BRCC36 protects CRBN from degradation by cleaving K63-linked polyubiquitin chains (Figure 4C) [49]. The compound SHIN1 up-regulates BRCC36 by binding to SHMT2, increasing CRBN levels and affecting lenalidomide sensitivity in MM cells.

Katia et al. found that blocking the transient receptor potential vanilloid receptor 1 (TRPV1) affects MM drug resistance [115]. The TRPV1 antagonist AMG9810 inhibits MM cell viability and synergizes with bortezomib. AMG9810 inhibits CXCR4 expression and activity, overcoming matrix-mediated drug resistance and regulating the ubiquitin signaling pathway, down-regulating 38 proteins related to ubiquitination during bortezomib/AMG9810 co-treatment and abolishing bortezomib-induced ubiquitination of cytoplasmic and mitochondrial proteins, thus altering drug targets and resistance [115]. IMiD agents suppress MM cell growth by CRBN-dependent ubiquitination and degradation of IKZF1/3. However, reduced CRBN expression or mutations impair this process, leading to IMiD resistance [30]. In contrast to IMiDs, GCK inhibitors overcome IMiD resistance by inducing ubiquitination and degradation of key transcription factors IKZF1/3 through a CRBN-independent mechanism [29].

#### 4.2.4. Deubiquitination

The reversal of ubiquitination by deubiquitinating enzymes (DUBs) is also implicated in MM drug resistance. High expression of DUBs such as Usp9x, Usp24, and Usp7 is associated with poor prognosis and may affect drug efficacy by stabilizing key pro-survival proteins like Mcl-1 [52]. The deubiquitinase USP7 interacts with NEK2 (a key regulator of chromosomal instability) and inhibits its ubiquitination, thereby stabilizing NEK2 protein levels. Overexpression of both USP7 and NEK2 in MM cells correlates with chemotherapy resistance and poor prognosis [51]. Notably, TRIP13 enhances USP7-mediated deubiquitination of NEK2 by forming a complex with USP7, which further stabilizes NEK2 and promotes resistance to PIs [88]. Additionally, USP7 stabilizes tumor suppressors like PTEN and p53, while TRIP13 modulates their subcellular localization, collectively contributing to altered drug responses [88]. CRBN downregulation or mutations prevent IMiDs from effectively recruiting IKZF1/3 to the E3 ubiquitin ligase complex, thereby blocking their degradation. This failure to degrade IKZF1/3 results in an inability to suppress c-Myc and interferon regulatory factor-4 (IRF4), ultimately driving IMiD resistance [84].

#### 4.2.5. Glycosylation

Aberrant glycosylation also affects MM drug resistance. Bortezomib-resistant U266-R cells exhibit higher levels of glycosylated uridine diphosphate (UDP) derivatives and increased activity of the hexosamine biosynthesis pathway (HBP) compared to bortezomib-sensitive U266-S cells (Figure 4D) [115]. HBP regulates protein O- and N-glycosylation and mitochondrial function. O-linked N-acetylglucosamine transferase (OGT), involved in glycosylation, is more highly expressed in U266-R, while O-GlcNAcase (OGA), involved in deglycosylation, is less expressed. After bortezomib treatment, U266-R maintains higher concentrations of glycosylated substrates, unlike U266-S, which may alter drug targets and resistance.

#### 4.2.6. Neddylation

The inhibition of the NEDD8-activating enzyme (NAE) blocks the activity of CRL (Cullin-RING ubiquitin ligase), leading to the stabilization and upregulation of the REDD1 protein. MLN4924, a NAE inhibitor, disrupts CRL-mediated ubiquitination by blocking neddylation, resulting in the accumulation of CRL substrates (e.g., p27, CDT1, NRF2) and REDD1 stabilization. REDD1 inhibits the PI3K/AKT/mTOR signaling pathway, thereby counteracting growth factor-mediated survival signals (e.g., IL-6, IGF-1) [91]. Importantly, MLN4924 demonstrates efficacy in bortezomib-resistant MM cells (e.g., ANBL-6-VSR), suggesting that its mechanism is independent of proteasome inhibition [116].

### 4.3. Regulating Cell Death and Survival Signals

The UPR is a cellular adaptive mechanism that activates in response to ER stress, enabling cells to mitigate proteotoxicity and maintain survival under drug-induced pressure. Moderate UPR activation helps alleviate ER stress caused by excessive protein synthesis, such as the production of monoclonal immunoglobulins in MM cells. However, sustained or overwhelming UPR activation can trigger cell death through JNK- or ER stress-dependent apoptotic pathways. MM cells initially rely on UPR to manage proteotoxic stress, making them sensitive to proteasome inhibitors (PIs). Over time, drug resistance develops due to reduced expression of key UPR regulators (e.g., XBP1), highlighting the dual role of UPR in MM therapy [117].

#### 4.3.1. Phosphorylation

**Figure 5:**
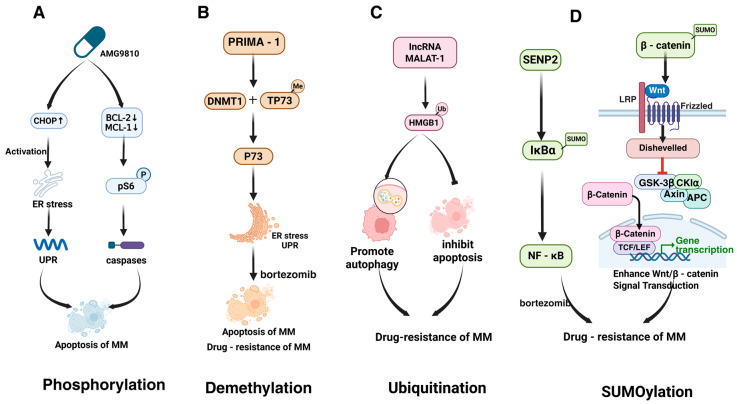
PTMs regulate cell death and survival in MM. This figure highlights key PTMs that regulate cell death and survival signals in MM. (**A**) Phosphorylation activates CHOP1 and ER stress pathways, inducing apoptosis. (**B**) Demethylation by PRIMA-1 depletes DNMT1 and restores sensitivity in bortezomib-resistant cells. (**C**) Ubiquitination of HMGB1, induced by lncRNA MALAT-1, promotes autophagy and inhibits apoptosis. (**D**) SUMOylation of IκBα and β-catenin activates NF-κB and Wnt/β-catenin signaling, reducing apoptosis and promoting drug resistance. The figure was created in Biorender. Hu, S. (2025) https://BioRender.com/ (accessed on 19 March 2025).

Phosphorylation modifications exert a core role in drug resistance in MM by regulating multiple key signaling pathways (Figure 5A). AMG9810 enhances the sensitivity of MM cells to drugs and overcomes resistance by upregulating C/EBP homologous protein (CHOP) to induce ER stress, triggering the UPR [115]. Concurrently, it reduces the expression of anti-apoptotic proteins BCL-2 and MCL-1 in a dose-dependent manner, inhibits phosphorylation of the mTOR downstream target pS6, and activates executor caspase-3 [118]. On the other hand, GCK (MAP4K2) drives cell proliferation and adaptive resistance by phosphorylating and activating the MKK4/7-JNK pathway, sustaining RAS mutation-dependent MAPK signaling hyperactivation. This phosphorylation-dependent MAPK overactivation not only promotes survival but may also enhance chemotherapy resistance by regulating downstream transcription factors such as c-JUN [29].

The phosphorylation status of the Akt/mTOR signaling pathway exhibits dual regulatory properties in MM drug resistance [119]. INPP4B enhances the efficacy of bortezomib by suppressing phosphorylation of Akt at Ser473, thereby attenuating PI3K/Akt/mTOR pathway activity, whereas loss of INPP4B leads to Akt hyperactivation and drug resistance [120,121,122]. Simultaneously, Akt-mediated phosphorylation inactivates EZH2, reducing H3K27me3 levels and derepressing pro-survival genes. This results in sustained expression of anti-apoptotic genes (e.g., BCL-2 family members), promoting cell adhesion-mediated drug resistance (CAM-DR) [61,123,124]. The crosstalk between epigenetic regulation and phosphorylation modifications underscores the complexity of signal integration within the tumor microenvironment.

Phosphorylation-driven activation of the NF-κB and STAT3 pathways further expands the network dimensions of resistance mechanisms. Docosahexaenoic acid (DHA) and eicosapentaenoic acid (EPA) activate the NF-κB pathway by increasing phosphorylation of its subunit p65 at Ser536, enhancing anti-apoptotic capacity and reducing sensitivity to bortezomib [125]. However, the timing of pre-treatment with DHA/EPA enhances toxicity, while co-administration causes antagonism—highlighting the critical role of temporal regulation. Additionally, JAK kinase promotes phosphorylation of STAT3 at Tyr705, upregulating anti-apoptotic targets such as BCL-XL and MCL-1, thereby constructing multilayered survival barriers for MM cells. These phosphorylation events collectively shape a dynamic signaling network in which hyperactivation of pro-survival pathways (e.g., Akt/mTOR, NF-κB, STAT3) synergizes with epigenetic regulation (e.g., EZH2 inactivation) to drive drug resistance [81,126].

#### 4.3.2. Demethylation

Small molecule PRIMA-1 was shown to influence drug resistance in MM through demethylation of p73. PRIMA-1 induces ER stress and the UPR by depleting DNA methyltransferase 1 (DNMT1), leading to TP73 demethylation and enhanced UPR [127]. Notably, PRIMA-1 exhibits greater toxicity in p53-deficient cells, suggesting that p73 may act as an alternative tumor suppressor. Knockdown of p73 attenuates PRIMA-1-induced apoptosis and ER stress marker upregulation, while p73 overexpression enhances UPR marker expression and growth inhibition (Figure 5B). PRIMA-1 also synergizes with bortezomib and partially restores sensitivity in bortezomib-resistant cells, potentially by amplifying UPR and modulating apoptotic pathways to overcome drug resistance [127].

#### 4.3.3. Ubiquitination

Zhuang et al. reported that ubiquitin-activating enzyme inhibitors can reverse MM resistance to PIs by inducing the UPR [127]. MM cells rely on UPR to alleviate ER stress caused by excessive M protein production, making them initially sensitive to PIs [117]. However, drug resistance development is associated with reduced expression of key UPR regulators, such as XBP1. Multiple PTMs of HMGB1 were also shown to promote MM drug resistance. HMGB1, which participates in DNA damage repair and autophagy, is highly expressed in MM and negatively correlates with patient survival (Figure 5C). For instance, lncRNA MALAT-1 induces HMGB1 ubiquitination at the post-translational level, increasing its expression, promoting autophagy, inhibiting apoptosis, and ultimately affecting drug sensitivity [87].

#### 4.3.4. Deubiquitination

USP14 and UCHL5 are highly expressed in MM and contribute to drug resistance by deubiquitinating misfolded or unfolded proteins, thereby reducing intracellular stress levels. Inhibition of these DUBs reduces MM cell viability, suppresses proliferation, and restores drug sensitivity in resistant cells [53]. Additionally, certain DUBs stabilize anti-apoptotic proteins like Mcl-1, modulating apoptotic pathways and influencing MM cell sensitivity to drugs [52].

#### 4.3.5. Glycosylation

Mitochondria-related changes have been implicated in MM drug resistance. For example, mitochondrial biogenesis markers PGC1α and SIRT1 are significantly upregulated in bortezomib-resistant U266-R cells compared to sensitive U266-S cells [88]. Notably, aberrant O-GlcNAcylation of mitochondrial proteins (e.g., VDAC1 and Complex I subunits) enhances oxidative phosphorylation efficiency, supporting energy demands in resistant cells [128]. U266-R cells also exhibit increased mitochondrial mass, elevated expression of the antioxidant enzyme GSTK1, and higher glutathione (GSH) concentrations. Furthermore, enhanced hexosamine biosynthesis pathway (HBP) activity in resistant cells promotes protein O-GlcNAcylation, which stabilizes anti-apoptotic BCL-2 family proteins and sustains mitochondrial membrane potential [129]. Unlike U266-S cells, which undergo mitochondrial depolarization and downregulation of cytochrome b and ATP synthase following bortezomib treatment, U266-R cells maintain mitochondrial integrity, suggesting that mitochondrial adaptations may reduce apoptosis and contribute to drug resistance [88].

#### 4.3.6. SUMOylation

Xie et al. demonstrated that downregulation of the SUMO-specific protease SENP2 in MM activates NF-κB by regulating the SUMOylation of IκBα, leading to bortezomib resistance [130]. Another study further highlighted that SUMOylation of β-catenin enhances Wnt/β-catenin signaling, promoting MM cell proliferation, reducing apoptosis, and impairing drug sensitivity (Figure 5D). Loss of CYLD stabilizes β-catenin and enhances β-catenin/TCF-mediated transcription, while BCL9 overexpression amplifies Wnt signaling, tumor cell proliferation, migration, and vascular endothelial growth factor (VEGF) expression, all of which may contribute to drug resistance [114].

#### 4.3.7. Deacetylation

Deacetylation (mediated by HDAC1) enhances the stability of HP1γ and strengthens its interaction with MDC1, promoting homologous recombination repair (HR) and improving cellular tolerance to DNA damage [95]. Concurrently, deacetylation induces HP1γ nuclear condensation through liquid-liquid phase separation (LLPS), which increases chromatin accessibility and upregulates the expression of drug resistance-related genes (e.g., CD40, FOS, JUN). These mechanisms collectively reduce sensitivity to PIs by enhancing DNA repair efficiency and activating survival pathways [95]

### 4.4. Modulating the Tumor Microenvironment and Immune Escape

#### 4.4.1. Sialylation

**Figure 6:**
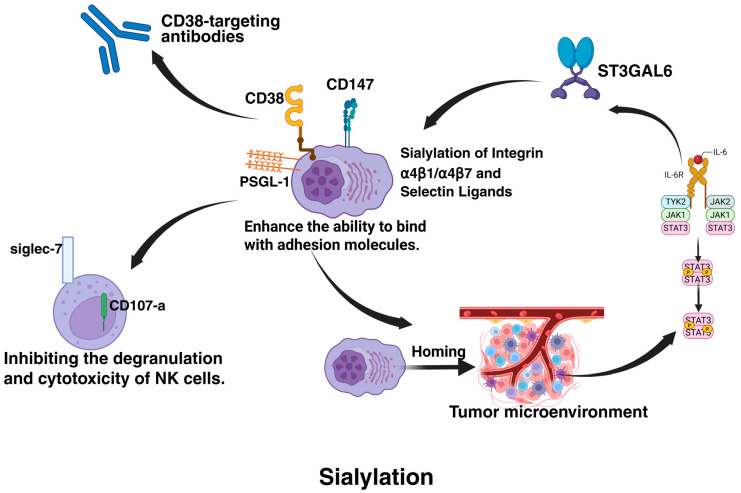
Sialylation-mediated regulation of the tumor microenvironment and immune escape in MM. This figure illustrates how sialylation promotes MM progression by enhancing tumor homing to the bone marrow microenvironment via integrin and selectin ligand modifications, reducing chemotherapeutic exposure and activating cell adhesion-mediated drug resistance (CAM-DR). Stromal cell-derived IL-6 activates STAT3, upregulating sialyltransferases (ST3GAL6/ST6GAL1) in a positive feedback loop, further driving sialylation (e.g., CD147) to enhance tumor proliferation. Sialylated PSGL-1 binds Siglec-7 on NK cells, suppressing cytotoxicity, while sialylation masks CD38 epitopes, impairing antibody recognition. The figure was created in Biorender. Hu, S. (2025) https://BioRender.com (accessed on 19 March 2025).

The drug resistance of MM is closely associated with sialyltransferase (STs)-mediated tumor cell-microenvironment interactions and immune escape (Figure 6). Studies demonstrate that STs enhance the binding capacity of MM cells to adhesion molecules (e.g., VCAM1, MADCAM1, and E-selectin) on bone marrow endothelial cells by catalyzing sialylation modifications of integrins α4β1/α4β7 and selectin ligands (e.g., SLea/x), thereby driving tumor cell homing to the bone marrow microenvironment (BMM) [92,93,94]. This homing process enables MM cells to acquire the protective barrier of BMM, which reduces exposure to chemotherapeutic agents (e.g., bortezomib) through physical isolation while activating cell adhesion-mediated drug resistance (CAM-DR). Notably, in a human MM mouse model, stromal cells in BMM activate the STAT3 signaling pathway via direct contact and secretion of factors such as IL-6, forming a positive feedback loop that persistently upregulates key enzymes like ST3GAL6 and ST6GAL1. This further promotes sialylation of membrane proteins such as CD147, enhancing tumor cell proliferation, metastasis, and microenvironmental adaptation [131].

At the immunomodulatory level, sialylation modifications establish a dual inhibitory barrier. On the one hand, sialylated PSGL-1 binds to the inhibitory receptor Siglec-7 on natural killer (NK) cells, significantly suppressing NK cell degranulation (reduced CD107a expression) and cytotoxicity (diminished IFN-γ release) [76]. On the other hand, sialylation-induced epitope masking of CD38 directly impairs the recognition efficiency of CD38-targeted antibodies like daratumumab, leading to resistance in immunotherapy. This immunosuppressive microenvironment can be reprogrammed through ST inhibitors [93]. The pan-sialyltransferase inhibitor 3F-Neu5Ac not only reduces sialylation levels of α4 integrins, weakening their ligand-binding affinity but also remodels immune cell composition—promoting infiltration of CD8^+^ T cells and NK cells while reducing regulatory T cell populations—thereby synergistically enhancing anti-tumor immune responses [131,132].

From a therapeutic perspective, 3F-Neu5Ac operates through two critical mechanisms: it inhibits ST3GAL6-catalyzed generation of E-selectin ligands, blocking MM cell homing to BMM and restricting tumor cell entry into drug sanctuaries; simultaneously, it disrupts glycosylation-dependent maturation of integrin α4 chains, directly impairing tumor-stromal cell adhesion-dependent survival signals [133]. In animal models, this dual action significantly enhances bortezomib sensitivity and prolongs survival [133]. However, it is noteworthy that this inhibitor fails to reverse IL-6-mediated stromal cell-induced resistance in vitro, suggesting that fully overcoming drug resistance requires combinatorial targeting of microenvironmental signaling pathways [131]. These findings provide a theoretical foundation for developing sialylation-targeted combination therapies, emphasizing the necessity to concurrently address intrinsic tumor cell resistance mechanisms and microenvironment-mediated extrinsic protective effects.

#### 4.4.2. Methylation

PRMT1 mediates methylation modifications that downregulate the expression of major histocompatibility complex class II (MHC II) genes, impairing the function of antigen-presenting cells and subsequently suppressing the expression of T cell activation markers CD25/CD69 [134]. This process contributes to the formation of an immunosuppressive microenvironment. Authors showed that inhibiting PRMT1 can restore MHC II-mediated antigen presentation efficiency, promote T cell activation, and provide a potential therapeutic target for reversing immune escape [134].

#### 4.4.3. Neddylation

Neddylation modifications regulate the activity of CRLs, the stability of the IMiD target CRBN, and the transcriptional network (IRF4/IKZF3/MEIS2), thereby influencing the immune escape capabilities and drug responses of MM cells and contributing to drug resistance. Inhibition of neddylation enhances immune recognition and restores IMiD sensitivity [71]. Additionally, TGFβ promotes immune escape and drug resistance in MM by suppressing the expression of natural killer (NK) cell activation receptors (e.g., NKG2D) and their effector functions (e.g., degranulation). Neddylation inhibition can partially reverse TGFβ-mediated suppression of NK cells, restoring NKG2D expression and perforin polarization, thereby enhancing NK cell-mediated killing of MM cells [71,116]. Furthermore, neddylation inhibition enhances the antibody-dependent cellular cytotoxicity (ADCC) effects mediated by daratumumab (anti-CD38) and elotuzumab (anti-SLAMF7) by upregulating Rac1/RhoA GTPase and F-actin levels in NK cells, improving immune synapse function, and overcoming resistance to monoclonal antibody therapy in MM [116].

## 5. Overcoming Drug Resistance in MM via Targeting PTMs

The critical role of PTMs in MM drug resistance highlights the potential for targeting these modifications to overcome resistance. Given the reversible nature of many PTMs and the availability of specific inhibitors, several strategies can be employed to modulate aberrant PTMs and restore drug sensitivity. This section recapitulates various approaches, including FDA-approved drugs, preclinical inhibitors, gene editing technologies, and novel therapeutic modalities like PROTACs and LYSOTACs, to target PTMs and overcome drug resistance in MM (Table 2).

**Table 2:** Strategies to Overcome Drug Resistance in MM by Targeting PTMs.

Strategy Category	Drug/Technology Name	Targeted PTM	Target/Pathway	Mechanism of Action	Ref
**FDA-Approved Drugs**					
Tyrosine Kinase Inhibitors (TKI)	Dasatinib	Phosphorylation	Src, BCR-ABL	Inhibits Src and BCR-ABL kinase activity, blocks PI3K/AKT and MAPK phosphorylation signaling, reverses resistance to PIs (PIs, e.g., bortezomib) and IMiDs (IMiDs, e.g., lenalidomide).	[135,136,137,138]
Tyrosine Kinase Inhibitors (TKI)	Imatinib	Phosphorylation	BCR-ABL	Binds specifically to the ATP-binding site of the BCR-ABL fusion protein, inhibits STAT5 and RAS/MAPK phosphorylation, and suppresses MM (MM) cell proliferation.	[135,136,137]
mTOR Inhibitor	Everolimus	Phosphorylation	mTORC1 (not mTORC2)	Inhibits mTORC1 phosphorylation activity, downregulates 4EBP1 and S6K1 signaling, blocks protein synthesis, and enhances sensitivity to PIs.	[135,136,137]
HDAC Inhibitor	Panobinostat	Acetylation, SUMOylation (indirect)	HDAC1/2/3/6	Inhibits HDAC6-mediated deacetylation of SUMOylation enzymes (e.g., SENP1), increases histone (H3K9/K14) and non-histone (e.g., HSP90) acetylation, and activates pro-apoptotic genes (BIM, NOXA).	[139,140,141,142,143]
HDAC Inhibitor	Vorinostat	Acetylation	HDAC1/2/3 (Class I)	Selectively inhibits Class I HDACs, enhances histone H3/H4 acetylation, promotes RelA acetylation to block its nuclear translocation, and reverses NF-κB-mediated drug resistance.	[141,142,143]
**Preclinical Inhibitors**					
SUMO E1 Inhibitor	TAK-981	SUMOylation	SUMO E1 enzyme (SAE1/SAE2)	Covalently inhibits SUMO activation, reduces SUMO modification of IRF4 and c-Myc, and enhances CRBN-dependent degradation induced by lenalidomide.	[90,144,145,146]
Neddylation Inhibitor	MLN4924	Ubiquitination (via Neddylation)	NEDD8-activating enzyme (NAE)	Inhibits neddylation of CRL complexes, leading to accumulation of pro-apoptotic proteins (NOXA, BIM) and stabilization of IκBα to suppress NF-κB signaling.	[69,147]
Glycosylation Inhibitor	OGT inhibitors (e.g., OSMI-1)	Glycosylation (O-GlcNAcylation)	O-GlcNAc transferase (OGT)	Reduces O-GlcNAc modification of β-catenin (Ser112) and c-Myc, inhibits Wnt/β-catenin and MYC signaling, and reverses bortezomib resistance.	[129,148]
**Gene Editing Technologies**					
CRISPR/Cas9	Ubc9/USP7 knockout	SUMOylation, Ubiquitination	Ubc9 (SUMO E2), USP7	Knockout of Ubc9 blocks SUMO-PML modification and inhibits DNA repair; knockout of USP7 stabilizes p53 and PTEN protein levels, inducing apoptosis.	[149,150,151,152,153,154,155]
siRNA/shRNA	SAE2/USP14 targeting	SUMOylation, Ubiquitination	SAE2, USP14	siRNA-mediated SAE2 silencing inhibits SUMO activation; shRNA-mediated USP14 silencing enhances ubiquitinated protein degradation and downregulates IRF4.	[156,157,158,159,160,161]
ASO	MDM2/USP7 targeting	Ubiquitination	MDM2, USP7	Antisense oligonucleotides (ASOs) suppress MDM2 or USP7 expression, stabilize ubiquitination levels of p53 or PTEN, and promote apoptosis.	[162,163]
**Novel Therapeutic Modalities**					
PROTACs	IRF4/c-Myc degraders	Ubiquitination	IRF4, c-Myc	Bifunctional molecules recruit CRBN or VHL to induce ubiquitination and degradation of IRF4 or c-Myc, directly eliminating drug resistance-associated transcription factors.	[164,165,166,167,168]
LYSOTACs	Wnt pathway-targeted degraders	Lysosomal degradation	LRP6, β-catenin	Target LRP6 or β-catenin for lysosomal degradation, inhibits Wnt signaling, and restore bortezomib sensitivity.	[169,170,171,172,173,174,175,176]
**Combination Therapies**					
SUMO inhibitor + PI	TAK-981 + Bortezomib	SUMOylation + Ubiquitination	SUMO pathway + Proteasome	TAK-981 induces misfolded protein accumulation, synergizing with bortezomib to activate the UPR (UPR) and ER stress, triggering apoptosis.	[70,111]
HDAC inhibitor + IMiD	Panobinostat + Lenalidomide	Acetylation + Ubiquitination	HDACs + CRBN	Panobinostat upregulates CRBN expression, enhancing lenalidomide-induced ubiquitination and degradation of IKZF1/3, thereby suppressing the IRF4-MYC axis.	[40,84,177]

### 5.1. FDA-Approved Drugs Targeting PTMs

FDA-approved tyrosine kinase inhibitors (TKIs) and mTOR inhibitors, such as dasatinib, imatinib, everolimus, and temsirolimus, target phosphorylation-dependent signaling pathways [135,136]. These drugs inhibit kinases like Src, BCR-ABL, and mTOR, which are often hyperactivated in drug-resistant MM. By modulating phosphorylation, these agents can restore sensitivity to PIs (PIs) and IMiDs (IMiDs) [137]. For example, dasatinib has shown potential in preclinical studies to overcome resistance by targeting phosphorylation-dependent survival pathways [136,138].

HDAC inhibitors, such as panobinostat, modulate acetylation levels of histones and non-histone proteins, influencing gene expression and protein function [139,140]. HDAC inhibitors have shown promise in overcoming resistance to PIs and IMiDs by restoring the expression of pro-apoptotic genes and enhancing the acetylation of drug targets. While no FDA-approved drugs specifically target SUMOylation, certain agents like HDAC inhibitors (e.g., panobinostat and vorinostat) can indirectly modulate SUMOylation pathways [141,142,143]. Panobinostat is approved for MM, inhibits HDACs, and has been shown to impact SUMOylation, potentially restoring sensitivity to PIs and IMiDs [143]. The repurposing of FDA-approved drugs to target PTMs offers a promising strategy for overcoming drug resistance in MM. Future research should focus on identifying novel PTM targets, developing more specific inhibitors, and exploring the potential of combination therapies.

### 5.2. Preclinical Inhibitors Targeting PTMs

Novel SUMOylation and ubiquitination inhibitors show promise in overcoming drug resistance in MM. SUMOylation inhibitors, such as TAK-981, a SUMO E1 inhibitor, have demonstrated potential in preclinical studies to increase sensitivity to lenalidomide by reducing the expression of key resistance regulators like IRF4 and c-Myc [90,178]. Similarly, neddylation-dependent ubiquitination inhibitors like MLN4924 (a NEDD8-activating enzyme inhibitor) have shown promise in preclinical models by blocking the activity of cullin-RING ligases (CRLs), essential for ubiquitination [69,147]. By inhibiting CRL activity, MLN4924 can induce the accumulation of pro-apoptotic proteins and enhance the efficacy of PIs. Additionally, targeting specific E3 ubiquitin ligases or deubiquitinases (DUBs) may provide more targeted approaches for overcoming resistance [45,104,179]. Glycosylation inhibitors also offer potential strategies to address drug resistance in MM [128]. Inhibitors of glycosylation enzymes, such as O-GlcNAc transferase (OGT) inhibitors, have shown potential in preclinical studies to restore drug sensitivity by modulating glycosylation levels [129,148]. Such small molecules are promising agents for overcoming drug resistance in MM.

### 5.3. Gene Editing Technologies

#### 5.3.1. CRISPR/Cas9

The CRISPR/Cas9 system has emerged as a powerful tool for precisely editing genes involved in PTMs, offering a direct approach to modulate key pathways MM [149]. By targeting enzymes central to SUMOylation, ubiquitination, or glycosylation, CRISPR/Cas9 can be used to either activate or inactivate specific PTMs, thereby modulating drug resistance pathways [150,151,152,153]. For instance, knocking out the SUMO E2 conjugating enzyme Ubc9 or the deubiquitinating enzyme USP7 has been shown to restore sensitivity to therapeutic agents by disrupting resistance mechanisms [154,155]. Additionally, CRISPR/Cas9 can be used to study the functional consequences of specific PTMs in MM, providing valuable insights into potential therapeutic targets and enabling the development of more effective treatments.

#### 5.3.2. Oligonucleotide-Based Interventions

Oligonucleotide-based strategies, such as RNA interference (RNAi) technologies, provide a powerful and versatile approach to selectively silence genes involved in PTMs (PTMs) [156,157,158]. RNAi tools, including small interfering RNA (siRNA) and short hairpin RNA (shRNA), can be designed to target and reduce the expression of key enzymes in PTM pathways, such as SUMOylation (e.g., SAE2) or deubiquitinating enzymes (e.g., USP14) [159,160,161]. By modulating these enzymes, RNAi can decrease specific PTMs and enhance drug sensitivity in cancer cells. The reversible and tunable nature of RNAi-based approaches allows researchers to precisely explore the roles of PTMs in drug resistance and identify potential therapeutic targets. This flexibility makes RNAi a valuable tool not only for preclinical studies but also for potential clinical applications in MM. Beyond RNAi, other oligonucleotide-based interventions, such as antisense oligonucleotides (ASOs), are also being explored for their ability to modulate gene expression and PTM-related pathways, further expanding the therapeutic potential of this approach [162,163].

### 5.4. Novel Therapeutic Modalities

#### 5.4.1. Proteolysis-Targeting Chimeras (PROTACs)

PROTACs are bifunctional molecules that recruit E3 ubiquitin ligases to target proteins for degradation [164,165]. By leveraging the ubiquitination pathway, PROTACs can selectively degrade proteins involved in drug resistance, such as IRF4 or c-Myc [166]. For example, a PROTAC targeting IRF4 could overcome lenalidomide resistance by reducing the levels of this transcription factor [167]. This approach offers a promising strategy for targeting undruggable proteins and overcoming resistance in MM. Recent studies have demonstrated the potential of PROTACs to enhance the efficacy of existing therapies by degrading key resistance proteins, thereby restoring sensitivity to treatments like PIs and IMiDs [165,168].

#### 5.4.2. Lysosome-Targeting Chimeras (LYSOTACs)

LYSOTACs represent a novel class of therapeutics that target proteins for lysosomal degradation [169,170]. Unlike PROTACs, which rely on the UPS, LYSOTACs utilize the lysosomal pathway to degrade target proteins. This approach could be particularly useful for targeting membrane-bound or extracellular proteins involved in drug resistance [169,170,171]. For instance, a LYSOTAC targeting components of the Wnt signaling pathway could reduce β-catenin levels and restore sensitivity to PIs [172,173,174]. By leveraging the lysosomal pathway, LYSOTACs offer a complementary strategy to PROTACs, expanding the range of targetable proteins and providing new opportunities to overcome resistance mechanisms in MM [175,176].

### 5.5. Combination Therapies

Combining PTM-targeting agents with existing therapies may provide a synergistic effect in overcoming drug resistance. For example, combining a SUMOylation inhibitor with a PI could enhance the accumulation of misfolded proteins and induce apoptosis in resistant MM cells [70,111]. Similarly, combining an HDAC inhibitor with an IMiD could restore the expression of pro-apoptotic genes and enhance the degradation of drug targets [84,177]. Rational combination therapies that target multiple PTMs simultaneously may offer a more effective strategy for overcoming resistance in MM. By addressing multiple resistance pathways, these combinations could provide a more comprehensive and durable therapeutic effect.

### 5.6. Personalized Therapy

The current treatment landscape for multiple myeloma remains largely rooted in a “one-size-fits-all” approach despite overwhelming evidence of the disease’s molecular and clinical heterogeneity. This paradigm fails to address critical variations in PTM-driven resistance mechanisms across patients, often resulting in suboptimal outcomes. As we enter the era of precision medicine, targeting individual patients’ unique PTM signatures—from phosphorylation patterns to glycosylation profiles—represents a transformative opportunity to overcome therapeutic resistance.

#### 5.6.1. Personalized CAR-T Therapy

BCMA, a critical target of CAR-T therapy, exhibits membrane surface expression regulated by both glycosylation and proteolytic cleavage. Research indicates that N-glycosylation modifications of BCMA significantly impact CAR-T cell recognition efficiency toward its epitopes, with specific glycosylation patterns potentially serving as biomarkers to predict CAR-T efficacy [180,181]. Notably, ADAM10 and γ-secretase-mediated proteolytic cleavage of BCMA releases soluble BCMA (sBCMA), resulting in antigen loss and immune evasion [3,182]. Clinical data demonstrate that dynamic sBCMA levels correlate strongly with tumor burden and prognosis (hazard ratio [HR] = 2.1, *p* < 0.001) [182]. Combining γ-secretase inhibitors (e.g., LY3039478) reduces sBCMA production, enhancing CAR-T cell targeting efficiency for membrane-bound BCMA by 40% [182].

#### 5.6.2. Targeted Interventions in Epigenetic Modifiers and Personalized Therapy

The t(4;14) translocation upregulates the MMSET (WHSC1) gene, leading to aberrant histone methylation modifications and conferring resistance to proteasome inhibitors [183]. Preclinical studies demonstrate that small-molecule inhibitors targeting MMSET can enhance sensitivity to bortezomib [184,185]. HDAC inhibitors, such as panobinostat, when combined with proteasome inhibitors, significantly prolonged PFS in relapsed/refractory patients in the phase III PANORAMA-1 trial (median PFS 12 vs. 8.1 months, *p* = 0.01) [182,186,187,188]. While challenges remain in standardizing PTM assessment and validating predictive algorithms, the field is moving decisively toward a future where MM treatment is tailored not just to cytogenetic risk groups but to each patient’s molecular vulnerability landscape.

## 6. The Challenges in Targeting PTMs for Therapy

### 6.1. Complexity and Dynamic Nature of PTMs

PTMs, including phosphorylation, acetylation, ubiquitination, glycosylation, and palmitoylation, are essential for regulating protein function, stability, and localization. In MM, abnormal PTM patterns can drive disease progression and affect treatment responses. However, the complexity of these modifications arises from their context-dependent roles, where different cell types, proteins, and disease states can influence how PTMs behave [15,189]. Importantly, crosstalk between different PTMs adds another layer of complexity. Lysine residues emerge as particularly contested sites, serving as substrates for acetylation, ubiquitination, SUMOylation, methylation, and other modifications [190,191]. This molecular “crowding” creates several layers of biological complexity: (1) Direct competition between modifications—for instance, acetylation and ubiquitination often vie for the same lysine, with acetylation typically stabilizing proteins while ubiquitination marks them for degradation; (2) Sequential dependencies where one modification enables another—phosphorylation frequently creates docking sites for E3 ubiquitin ligases, coupling signaling to protein turnover; (3) Bistable switches where mutually exclusive modifications create digital regulatory nodes [189]. The dynamic nature makes it challenging to target a specific PTM without inadvertently disrupting other critical cellular functions. For example, targeting the enzymes responsible for phosphorylation or acetylation might alter multiple downstream pathways, complicating the therapeutic strategy. This requires developing therapies that balance efficacy while minimizing unintended effects in non-cancerous tissues. Recent advances in structural biology and covalent inhibitor design are beginning to meet this challenge, as demonstrated by the development of lysine-targeting warheads that can distinguish between acetylation and ubiquitination sites based on local conformational features.

### 6.2. Heterogeneity and Plasticity of MM Cells

The heterogeneity and plasticity of cancer cells present significant barriers to PTM-targeted therapies in MM [16,192,193,194]. Different patients may exhibit distinct PTM patterns, even within the same subtype of MM, leading to varied responses to treatment. Furthermore, cancer cells can adapt by changing their PTM profiles in response to therapy, leading to treatment resistance [111,195,196,197]. This variability requires personalized therapeutic approaches that consider each patient’s unique PTM landscape, as a one-size-fits-all treatment is unlikely to be effective across the diverse patient population seen in MM.

### 6.3. Technological Limitations

The clinical translation of PTM-targeted therapies is hindered by the limitations of current technologies used to detect and analyze PTMs. Although advancements in proteomics have enhanced our ability to map PTM landscapes, these methods are not yet fully optimized for routine clinical use [198,199]. In MM, identifying PTM biomarkers that correlate with disease progression or therapeutic response is crucial to advancing these therapies. Additionally, rigorous clinical trials are required to ensure the safety and efficacy of PTM-targeted treatments, given the complexity of PTMs in cancer biology and their involvement in broader regulatory networks.

While PTMs offer promising therapeutic targets in MM, addressing the complexity, heterogeneity, and technological limitations associated with these modifications is essential for translating PTM-targeted therapies into clinical practice. Future research should focus on developing more precise and personalized approaches to overcome these challenges and improve outcomes for patients with MM.

## 7. Concluding Remarks

MM remains a challenging hematologic malignancy, primarily due to the inevitable development of therapeutic resistance. PTMs, including phosphorylation, acetylation, ubiquitination, and glycosylation, play pivotal roles in regulating protein function, stability, and cellular interactions. Dysregulation of these modifications contributes significantly to MM pathogenesis, tumor progression, and the emergence of drug resistance, particularly in relapsed or refractory cases. Understanding the molecular mechanisms underlying PTM-mediated resistance is essential for developing effective therapeutic strategies.

This review highlights the critical roles of PTMs in driving drug resistance in MM. Dysregulated PTMs—including phosphorylation, acetylation, ubiquitination, and glycosylation—alter key signaling pathways (e.g., NF-κB, STAT3, Wnt/β-catenin), metabolic reprogramming, and interactions with the tumor microenvironment, enabling MM cells to evade therapies such as PIs and IMiDs. Notably, PTM-mediated mechanisms mainly include modulation of drug uptake/efflux, altering drug targets, regulating cell death and survival, and immune evasion. Emerging strategies, such as HDAC inhibitors, SUMOylation blockers, and ubiquitin-proteasome modulators, demonstrate preclinical success in reversing resistance. However, challenges persist due to PTM crosstalk, tumor heterogeneity, and adaptive responses in the bone marrow niche.

To translate these insights into clinical breakthroughs, future efforts should prioritize (1) Mechanistic studies to decode context-dependent PTM interactions (e.g., SUMOylation-ubiquitination competition) using single-cell proteomics; (2) Biomarker development via high-throughput PTM profiling to stratify patients for targeted therapies; (3) Innovative therapeutics, including isoform-specific PTM inhibitors (e.g., HDAC6-selective agents) and combination regimens (e.g., neddylation inhibitors + anti-CD38 antibodies); and (4) Microenvironmental targeting to disrupt PTM-driven stromal protection (e.g., CRISPR-edited CAR-T cells against glycosylated BCMA). Collaborative interdisciplinary research—integrating epigenomics, structural biology, and AI-driven drug design—will be essential to overcome resistance and achieve durable remissions in MM.

In conclusion, this review underscores PTMs as central players in MM drug resistance and advocates for their therapeutic targeting. By integrating mechanistic insights with innovative technologies, we envision a paradigm shift toward precision medicine in MM, where PTM modulation could transform relapsed/refractory cases into manageable chronic conditions. Future research must bridge the gap between molecular understanding and clinical application to realize this potential.

## Data Availability

Not applicable.

## Abbreviations

ABC	ATP Binding Cassette
ASOs	Antisense Oligonucleotides
BER	Base Excision Repair
BiTEs	Bispecific T-Cell Engagers
BCMA	B Cell Maturation Antigen
BMM	Bone Marrow Microenvironment
CAM DR	Cell Adhesion-Mediated Drug Resistance
CAR-T	Chimeric Antigen Receptor T-Cell
CHOP	C EBP Homologous Protein
CIN	Chromosomal Instability
CRBN	Cereblon
CRLs	Cullin-RING Ligases
DHA	Docosahexaenoic Acid
DUBs	Deubiquitinating Enzymes
EPA	Eicosapentaenoic Acid
ER	Endoplasmic Reticulum
GWAS	Genome-Wide Association Studies
HATs	Histone Acetyltransferases
HBP	Hexosamine Biosynthesis Pathway
HDACs	Histone Deacetylases
HIF-1α	Hypoxia Inducible Factor 1 Alpha
IMiDs	Immunomodulatory Drugs
IRF4	Interferon Regulatory Factor 4
ISS	International Staging System
LLPS	Liquid-Liquid Phase Separation
LYSOTACs	Lysosome-Targeting Chimeras
mAbs	Monoclonal Antibodies
MDR1	Multidrug Resistance Protein 1
MM	Multiple Myeloma
mTOR	Mammalian Target of Rapamycin
NAE	NEDD8-Activating Enzyme
NF-κB	Nuclear Factor Kappa-Light-Chain-Enhancer of Activated B Cells
OGT	O-GlcNAc Transferase
OS	Overall Survival
PFS	Progression-Free Survival
PIs	Proteasome Inhibitors
PROTACs	Proteolysis-Targeting Chimeras
PTMs	Post Translational Modifications
RRMM	Relapsed Refractory Multiple Myeloma
SENPs	SUMO-Specific Proteases
SNAC	S-Nitroso-N-Acetylcysteine
SOCS1	Suppressor of Cytokine Signaling 1
STAT3	Signal Transducer and Activator of Transcription 3
STs	Sialyltransferases
TKIs	Tyrosine Kinase Inhibitors
TRPV1	Transient Receptor Potential Vanilloid 1
UCHs	Ubiquitin C-Terminal Hydrolases
UPR	Unfolded Protein Response
UPS	Ubiquitin-Proteasome System
USPs	Ubiquitin-Specific Proteases
Wnt	Wingless Integrated Signaling Pathway
